# Blind Frequency Estimation and Symbol Recovery for the Analytically Solvable Chaotic System

**DOI:** 10.3390/e21080791

**Published:** 2019-08-13

**Authors:** Ang Zhou, Shilian Wang, Junshan Luo

**Affiliations:** College of Electronic Science, National University of Defense Technology, Changsha 410073, China

**Keywords:** analytically solvable chaotic system, frequency estimation, performance evaluation

## Abstract

The analytically solvable chaotic system (ASCS) is a promising chaotic system in chaos communication and radar fields. In this paper, we propose a maximum likelihood estimator (MLE) to estimate the frequency of ASCS, then a difference-integral (DI) detector is designed with the estimated frequency, and the symbols encoded in the signal are recovered. In the proposed method, the frequency parameter is estimated by an MLE based on the square power of the received signal. The Cramer-Rao lower bound in blind frequency estimation and the bit error performance in symbol detection are analyzed to assess the performance of the proposed method. Numerical results validate the analysis and demonstrate that the proposed symbol detector achieves the error performance with a little cost of 1 dB compared to the coherent detector. The robustness of the proposed method towards parameters is also verified through simulations.

## 1. Introduction

The characteristics of a chaotic signal—such as noise-like obscurity, aperiodicity, thumbtack-like correlation, sensitivity to initial conditions and system parameters—enable a chaotic signal to be widely investigated in engineering applications. Specifically, the noise-like property that determines the chaotic signal involves a low probability of interception (LPI), which is essential for secure communication and radar applications.

In the last decade, a class of analytically solvable chaotic system (ASCS) was introduced [1,2,3]. ASCS is defined by a second-order differential equation and a discrete switching condition controlled by binary symbols. The ASCS can be expressed analytically with an exact solution by solving the differential equation. It can also be described by a linear convolution with a fixed basis function and binary symbols. Due to the fixed shape of the basis function, a matched filter was then proposed to recover the binary symbols from the received signal [4]. Experiment and analysis proved that its bit error rate (BER) performance is comparable to that of the binary phase-shift keying (BPSK). Moreover, the circuit implementation of ASCS and the corresponding matched-filter based detector were provided [2]. For its excellent performance against noise and its simplicity in circuit realization, ASCS has been used in chaos communication [5,6,7,8,9,10,11], chaos radar [12,13], and underwater ranging and navigation [14]. Reference [6] proved that the information encoded in the chaotic signal is kept after being transmitted through a wireless channel with multipath effects. In Reference [8], an easy coding method was proposed to encode the information bits into the initial value of the ASCS oscillator. The application of ASCS to the wireless channel was analyzed and tested through a circuit implementation. In Reference [9], a high-frequency reverse-time ASCS was successfully generated in an optical carrier, which is promising for optical secure communication. In Reference [11], a resisting-multipath technique was proposed to tackle the inter-symbol interference caused by multipath. In Reference [10], an underwater spread spectrum scheme based on ASCS was proposed to provide superior reliability and security compared with the conventional chaos direct spread spectrum system.

Although ASCS shows superior performance in communication and radar applications, the ASCS signal can be considered as simply a BPSK communication waveform with an unconventional basis function so that it has no inherent security. The dynamical characteristics and the exact solution of such a class of ASCS signal make it possible to incoherently recover the symbols conveyed in the chaotic signal even if it is severely contaminated by noise. In this paper, we propose an efficient method for estimating the frequency of ASCS, and for detecting the symbols encoded in the waveform. The system parameters of ASCS and the binary symbols encoded in the continuous chaotic waveform can be estimated directly from the received signal via a difference-integral (DI) detector. For a potential attacker, this method process requires only the knowledge of the form of ASCS, which is practical in implementation.

Based on the square power spectrum of the received signal, we first proposed a maximum likelihood estimator (MLE) to obtain the frequency parameter of the ASCS. For the symbol detection, we proposed a DI detector in which the binary symbols are retrieved by cumulatively summing the difference between the received signal and its time-delayed version. This detector requires no parameters of the transmitter other than the basis frequency, which can be estimated. The advantages of the proposed method are two-fold: first, it does not require prior knowledge at the transmitter other than the form of ASCS; second, although the error performance of the proposed symbol detector is around 1 dB worse than that of the coherent detector for ASCS, it provides a new tool for a potential adversary hoping to incoherently detect an ASCS signal.

The rest of this paper is organized as follows: In Section 2, we provide a brief review of the system description and exact solution of ASCS. In Section 3, the MLE based on the square power spectrum is proposed to estimate the basis frequency of ASCS. The performance of the estimator is also evaluated. In Section 4, a DI detector is proposed with detailed theoretical derivations. In Section 5, a closed-form expression for error probability in symbol detection is derived and verified through simulations. The robustness of the detector is also discussed. Finally, some conclusions are made in Section 6.

## 2. The Analytically Solvable Chaotic System

The standard form of ASCS is described with a differential equation as [1]

(1) $$\ddot{u}-2\beta \dot{u}+\left(\omega^{2}+\beta^{2}\right)\left(u-s\right)=0$$

where ω determines the symbol period as T=2π/ω and $\beta \in (0,T^{-1}\ln2)$ is the damping coefficient of the linear part of ASCS system. The waveform u(t)∈R is continuous and state s∈{±1} is discrete. The state can be controlled by a binary symbol sequence s(t) which is set as the sign of current u(t) once the switching condition meets and is held constant until the next time when the switching condition triggers. The switching condition and state change process are described as

(2) $$\dot{u}=0\Rightarrow s\left(t\right)=\mathrm{sgn}\left\{u\left(t\right)\right\}.$$

**Figure 1:**
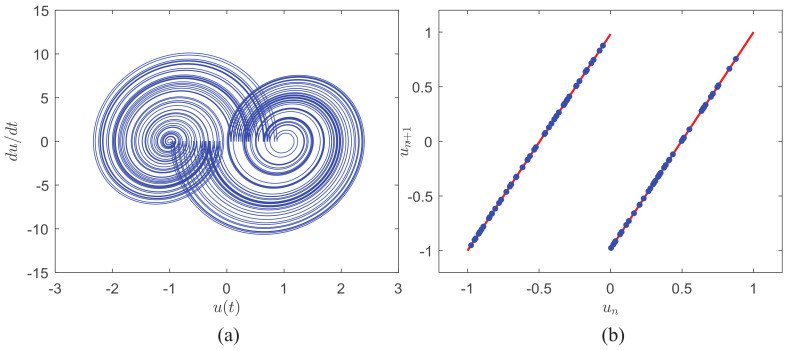
The chaos feature of ASCS. (**a**) The phase-space projection. (**b**)The successive return map with the standard iteration relation lines.

The chaotic signal u(t) can be obtained by the numerical solution of the Equation (1) with an adjustable step size Runge-Kutta integrator. Suppose the initial conditions are |u(0)|<1 and $\dot{u}\left(0\right)=0$, the exact analytic local solution of Equation (1) for the segment of nT≤t<(n+1)T is derived as

(3) $$u\left(t\right)=s_{n}+\left(u_{n}-s_{n}\right)e^{\beta (t-nT)}\left(\cos\omega t-\frac{\beta }{\omega }\sin\omega t\right)$$

with the iteration relation $u_{n+1}=e^{2\beta \pi /\omega }u_{n}-\left(e^{2\beta \pi /\omega }-1\right)s_{n}$, where *n* is an integer. Figure 1 shows the corresponding phase-space projection and the return map of the iterated shift relation at regular return times nT where *n* is an integer. The system parameters are T=1 and β=ln2. As Figure 1a suggests, ASCS has an attractor topologically similar to the well-known Lorenz attractor. As Figure 1b shows, the sampled values at nT are divided into two groups by the binary symbol sequence s(t) and follow the standard iteration relation lines with the fixed slope of $e^{2\beta \pi /\omega }$. Hence, the piecewise linear return map implies the dynamical system generated from Equation (1) is chaotic with Lyapunov exponent λ=β.

Based on the iteration relation of ASCS, the *N*th return point $u_{N}$ and its former symbols determine the initial value $u_{0}$, which is given by

(4) $$u_{0}=e^{-N\beta T}u_{N}+\left(1-e^{-\beta T}\right)\sum_{m=0}^{N-1}s_{m}e^{-m\beta T}.$$

From the analytic solution Equation (3), u(t) can be derived as a linear convolution of a basis function and a binary symbol sequence when *N* approaches to infinity:

(5) $$u\left(t\right)=\sum_{m=-\infty }^{N}s_{m}P\left(t-mT\right)$$

where *m* is an integer and the basis function P(t) is defined as

(6) $$\begin{array}{c} P\left(t\right)=\left\{\begin{array}{cc} \left(1-e^{-\beta T}\right)e^{\beta t}\left(\cos\omega t-\frac{\beta }{\omega }\sin\omega t\right), & t<0\\ 1-e^{\beta (t-T)}\left(\cos\omega t-\frac{\beta }{\omega }\sin\omega t\right), & 0\leq t<T\\ 0, & T\leq t. \end{array}\right. \end{array}$$

**Figure 2:**
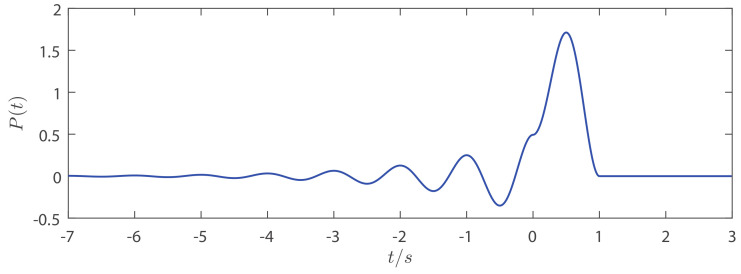
The basis function of the analytically solvable chaotic system (ASCS) for T=1 s and β=ln2.

Figure 2 shows the shape of the basis function P(t) for T=1 and β=ln2. As Equation (5) implies, the continuous waveform of u(t) can be generated by the sum of binary symbols and the time delayed basis function P(t). Thus, binary symbols can be encoded into the continuous chaotic waveform u(t).

## 3. Frequency Estimation for Chaotic Signal

In ASCS, the frequency parameter ω determines the symbol rate, which is significantly important in symbol detection. In the following, we derive the amplitude spectrum of the square power of u(t) and then propose a frequency estimator based on it.

### 3.1. Amplitude Spectrum of the Square Power of the Chaotic Signal

Assume the basis radian frequency of ASCS is $\omega_{0}$. Based on the analytical solution Equation (3), the local waveform of u(t) for mT≤t≤(m+1)T is

(7) $$u\left(t\right)=s_{m}+D_{m}e^{\beta (t-mT)}A\cos\left(\omega_{0}t+\varphi \right)$$

where $D_{m}=u_{m}-s_{m}$; $A=\sqrt{1+\beta^{2}/\omega_{0}}$; $\varphi =\arctan\beta /\omega_{0}$. The feature of the amplitude spectrum of the ASCS has been calculated and analyzed in References [4,15]. The spectrum of the chaotic waveform u(t) shows zeros at the fundamental frequency and its harmonics. Although this conclusion is determined, the frequency component with zero amplitude is difficult to locate accurately as the zero zones are often too vague to detect, especially when the signal-to-noise-ratio (SNR) is low. In the following, we derive the amplitude spectrum of the square power of u(t) and then propose a frequency estimator based on it.

The square power of u(t) is

(8) $$v\left(t\right)=s_{m}^{2}+2s_{m}D_{m}e^{\beta (t-mT)}A\cos\left(\omega_{0}t+\varphi \right)+\frac{1}{2}D_{m}^{2}A^{2}e^{2\beta (t-mT)}\left(\cos\left(2\omega_{0}t+2\varphi \right)+1\right).$$

Since $s_{m}^{2}=1$ is constant and contributes to the spike in the zero frequency component, we subtract it from v(t) and have w(t)=v(t)−1 as

(9) $$w\left(t\right)=w_{1}\left(t\right)+w_{2}\left(t\right)$$

where $w_{1}\left(t\right)=2s_{m}D_{m}e^{\beta (t-mT)}A\cos\left(\omega_{0}t+\varphi \right)$; $w_{2}\left(t\right)=\frac{1}{2}D_{m}^{2}A^{2}e^{2\beta (t-mT)}\left(\cos\left(2\omega_{0}t+2\varphi \right)+1\right)$. Then we derive the Fourier transform of each part. The Fourier transform for $w_{1}\left(t\right)$ is

(10) $$\begin{array}{cc} W_{1}\left(\omega \right) & =\int_{-\infty }^{\infty }w_{1}\left(t\right)e^{-j\omega t}dt\\ & =2A\sum_{m=-\infty }^{\infty }s_{m}D_{m}\int_{mT}^{(m+1)T}e^{\beta (t-mT)}\cos\left(\omega_{0}t+\varphi \right)e^{-j\omega t}dt \end{array}$$

Since the integral is difficult to derive, we calculate the Fourier coefficients as

(11) $$a\left(\omega \right)=2A\sum_{m=-\infty }^{\infty }s_{m}D_{m}\int_{mT}^{(m+1)T}e^{\beta (t-mT)}\cos\left(\omega_{0}t+\varphi \right)\cos\omega tdt$$

and

(12) $$b\left(\omega \right)=2A\sum_{m=-\infty }^{\infty }s_{m}D_{m}\int_{mT}^{(m+1)T}e^{\beta (t-mT)}\cos\left(\omega_{0}t+\varphi \right)\sin\omega tdt.$$

By computing the integral, we have a(ω) as

$$\begin{array}{cc} a(\omega )= & A\sum_{m=-\infty }^{\infty }s_{m}D_{m}(\frac{-\beta \cos\theta_{m}-\left(\omega_{0}-\omega \right)\sin\theta_{m}}{\beta^{2}+{\left(\omega_{0}-\omega \right)}^{2}}-\frac{\beta \cos\lambda_{m}+\left(\omega_{0}+\omega \right)\sin\lambda_{m}}{\beta^{2}+{\left(\omega_{0}+\omega \right)}^{2}}\\ & +e^{\beta T}\left(\frac{\beta \cos\delta_{m}+\left(\omega_{0}-\omega \right)\sin\delta_{m}}{\beta^{2}+{\left(\omega_{0}-\omega \right)}^{2}}+\frac{\beta \cos\eta_{m}+\left(\omega_{0}+\omega \right)\sin\eta_{m}}{\beta^{2}+{\left(\omega_{0}+\omega \right)}^{2}}\right)) \end{array}$$

where $\varphi -mT\omega =\theta_{m}$; $\varphi +mT\omega =\lambda_{m}$; $\varphi -\left(1+m\right)T\omega =\delta_{m}$; $\varphi +\left(1+m\right)T\omega =\eta_{m}$. As *m* changes, the variables $\theta_{m}$, $\lambda_{m}$, $\delta_{m}$, and $\eta_{m}$ change gradually. Take the $\cos\theta_{m}$ as an example. Since $\theta_{m}$ is ergodic in −∞ to *∞* when $\omega \neq n\omega_{0}$(n=0,1,…,), then $\sum_{m=-\infty }^{\infty }\theta_{m}\to 0$. Hence, we have a(ω)≈0. When $\omega =n\omega_{0}$, however, the variables $\theta_{m}$, $\lambda_{m}$, $\delta_{m}$, and $\eta_{m}$ keep constant since mTω=2mnπ. The analysis of b(ω) is similar to that of a(ω) (see Equation (A1) in Appendix A). After obtaining the Fourier transform coefficients, the amplitude spectrum is then calculated by $|W_{1}\left(\omega \right)|=\sqrt{a^{2}\left(\omega \right)+b^{2}\left(\omega \right)}$ and the approximate result for $\omega =n\omega_{0}$ is

(13) $$|W_{1}\left(\omega \right)|=2AI_{1}\left(n\right)\left|\sum_{m=-\infty }^{\infty }s_{m}D_{m}\right|$$

where

(14) $$\begin{array}{c} I_{1}\left(n\right)=\sqrt{\frac{{\left(e^{\beta T}-1\right)}^{2}n^{2}\omega_{0}^{4}}{\left(\beta^{2}+\omega_{0}^{2}\right)\left(\beta^{2}+{\left(n-1\right)}^{2}\omega_{0}^{2}\right)\left(\beta^{2}+{\left(1+n\right)}^{2}\omega_{0}^{2}\right)}}. \end{array}$$

Since $s_{m}D_{m}$ is uniformly distributed in [−1,0], the average of $|W_{1}\left(\omega \right)|$ for $\omega =n\omega_{0}$ is fixed at $AI_{1}\left(n\right)$. The expression of $I_{1}\left(n\right)$ implies the existence of spikes at the integer multiplies of the basis frequency $\omega_{0}$ and the amplitude of the spikes shrinks as *n* increases. Hence, the single-side amplitude spectrum $|W_{1}\left(\omega \right)|$ shows the maximum spike at n=1.

The analysis of the amplitude spectrum of $w_{2}\left(t\right)$ is similar to the above and the exact analytic expression of $|W_{2}\left(\omega \right)|$ for $\omega =n\omega_{0}$ is given by

(15) $$|W_{2}\left(\omega \right)|=\frac{2A^{2}\left(e^{2\beta T}-\frac{1}{2}\right)}{\beta^{2}+\omega_{0}^{2}}I_{2}\left(n\right)\sum_{m=-\infty }^{\infty }D_{m}^{2}$$

where

(16) $$I_{2}\left(n\right)=\sqrt{\frac{100\beta^{4}\omega_{0}^{4}+8\left(5+2n^{2}\right)\beta^{2}\omega_{0}^{6}+{\left(n^{2}-2\right)}^{2}\omega_{0}^{8}}{\left(4\beta^{2}+{\left(n-2\right)}^{2}\omega_{0}^{2}\right)\left(4\beta^{2}+n^{2}\omega_{0}^{2}\right)\left(4\beta^{2}+{\left(2+n\right)}^{2}\omega_{0}^{2}\right)}}.$$

The detailed derivation is provided in Appendix A. Note that the random variable $D_{m}^{2}$ follows a uniform distribution in [0,1], hence the average of $|W_{2}\left(\omega \right)|$ is fixed at $\frac{A^{2}\left(e^{2\beta T}-\frac{1}{2}\right)}{\beta^{2}+\omega_{0}^{2}}I_{2}\left(n\right)$. As the expression of $I_{2}\left(n\right)$ implies, the spikes exist at the integer multiplies of the basis frequency. When n=0, the partial spectrum sees a maximum spike which is caused by the direct current component in $w_{2}\left(t\right)$. When n=1, the spectrum sees a second maximum spike.

**Figure 3:**
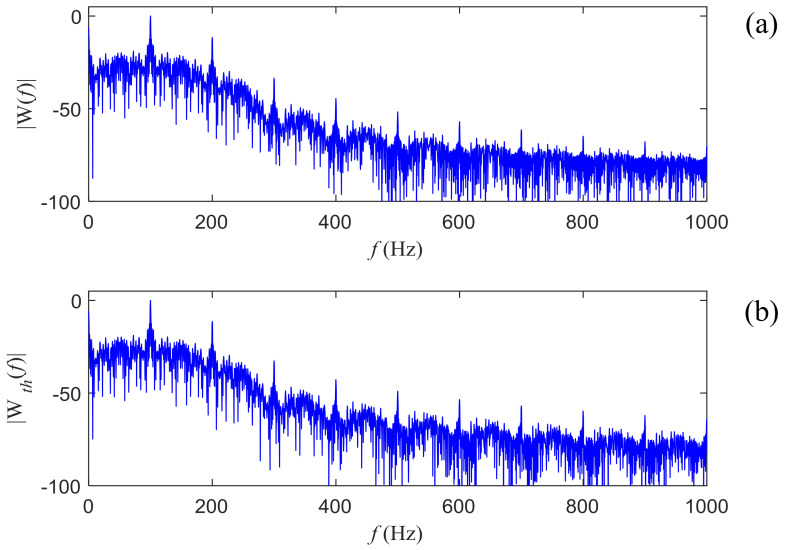
(**a**) The normalized amplitude spectrum obtained by FFT for the chaotic signal with the basis frequency of 100 Hz. (**b**) The normalized amplitude spectrum obtained by the theoretical Fourier transform for the same chaotic signal.

By summing the $|W_{1}\left(\omega \right)|$ and $|W_{2}\left(\omega \right)|$ together, we have the final amplitude spectrum of w(t). Figure 3a,b show a typical amplitude spectrum and the theoretical amplitude spectrum of the chaotic signal respectively, with the basis frequency $f_{0}$ as 100 Hz. From the comparison, the amplitude spectrum obtained by fast Fourier transform (FFT) matches the theoretical one well, proving the derivations above are reasonable and valid. The spikes occur at $nf_{0}$ (n = 1, 2, …), that is, the inter multiplies the basis frequency and shows a damping amplitude as *n* increases. Despite the zero frequency part, the maximum spike occurs exactly at 100 Hz and the second highest shows at 200 Hz. Obviously, the basis frequency can be estimated from the maximum spike in the amplitude spectrum of the square power of the chaotic signal.

### 3.2. Maximum Likelihood Estimator and Performance Analysis

Based on the analysis of the square power of the chaotic signal, we proposed a maximum likelihood estimator (MLE). Suppose the observed signal with a time length of MT is $r=(r_{0},r_{1},\ldots ,r_{N})$, with

(17) $$r_{k}=u_{k}+n_{k}$$

for k=0,1,2,…,N. The sample is $u_{k}=u\left(kf_{s}t\right)$ and $f_{s}$ is the sampling frequency; $N=MTf_{s}$ is the number of samples; $n_{k}$ is the additive Gaussian noise with zero mean and variance of $\sigma^{2}$. We calculate the square power of the observed signal and subtract its direct current component as

(18) $$x_{k}=u_{k}^{2}+2u_{k}n_{k}+n_{k}^{2}-E\left(r_{k}^{2}\right)$$

where the first term contains signal part while the second and the third term are noise-related. The last term $E(r_{k}^{2})$ is the average value of the square power of the received signal. Since the term $2u_{k}n_{k}$ has zero mean, we have $E\left(r_{k}^{2}\right)=E\left(u_{k}^{2}\right)+\sigma^{2}$. The basis frequency of the chaotic signal is estimated by an MLE as

(19) $${\hat{\omega }}_{MLE}=\underset{\omega }{\arg\max}{\left|\sum_{k=0}^{N-1}x_{k}e^{-j\omega_{k}}\right|}^{2}.$$

According to the Fourier transform of x(t), we have $E\left({\hat{\omega }}_{0\_MLE}\right)=\omega_{0}$. Hence, the estimator is unbiased. To assess whether the proposed unbiased estimator is asymptotically efficient or not, we continue to derive the Cramer-Rao lower bound (CRLB) in the following. Note that the segment of the first term $v_{k}=u_{k}^{2}$ in $x_{k}$ for $mTf_{s}\leq k<\left(m+1\right)Tf_{s}$ is

(20) $$\begin{array}{cc} v_{k}= & 1+2s_{m}D_{m}e^{\beta (\frac{k}{f_{s}}-mT)}A\cos\left(2\pi f_{0}\frac{k}{f_{s}}+\varphi \right)\\ & +\frac{1}{2}D_{m}^{2}A^{2}e^{2\beta (\frac{k}{f_{s}}-mT)}\left(\cos(4\pi f_{0}\frac{k}{f_{s}}+2\varphi )+1\right) \end{array}$$

where $D_{m}=u_{m}-s_{m}$. For simplicity in derivation, we assume the noise term distributes normally with zero mean and variance of $\sigma_{x}^{2}=2\sigma^{4}+2\sigma_{un}^{2}$, where $\sigma_{un}^{2}$ is the variance of the term $2u_{k}n_{k}$. Like the similar process in parameter estimation with white Gaussian noise, we have the Fisher information of the estimated frequency as

(21) $$I\left({\hat{f}}_{0}\right)=-\frac{1}{\sigma_{x}^{2}}\sum_{k=0}^{N-1}{\left(\frac{\partial v_{k}\left(f_{0}\right)}{\partial f_{0}}\right)}^{2}$$

where the cumulative sum part is derived as

(22) $$\begin{array}{cc} \sum_{k=0}^{N-1}{\left(\frac{\partial v_{k}\left(f_{0}\right)}{\partial f_{0}}\right)}^{2}= & \sum_{m=0}^{M-1}\sum_{k=mTf_{s}}^{\left(m+1\right)Tf_{s}-1}(-4\pi \frac{k}{f_{s}}s_{m}D_{m}e^{\beta (\frac{k}{f_{s}}-mT)}A\sin\left(2\pi f_{0}\frac{k}{f_{s}}+\varphi \right)\\ & -2\pi \frac{k}{f_{s}}D_{m}^{2}A^{2}e^{2\beta (\frac{k}{f_{s}}-mT)}\sin\left(4\pi f_{0}\frac{k}{f_{s}}+2\varphi \right){)}^{2}. \end{array}$$

**Figure 4:**
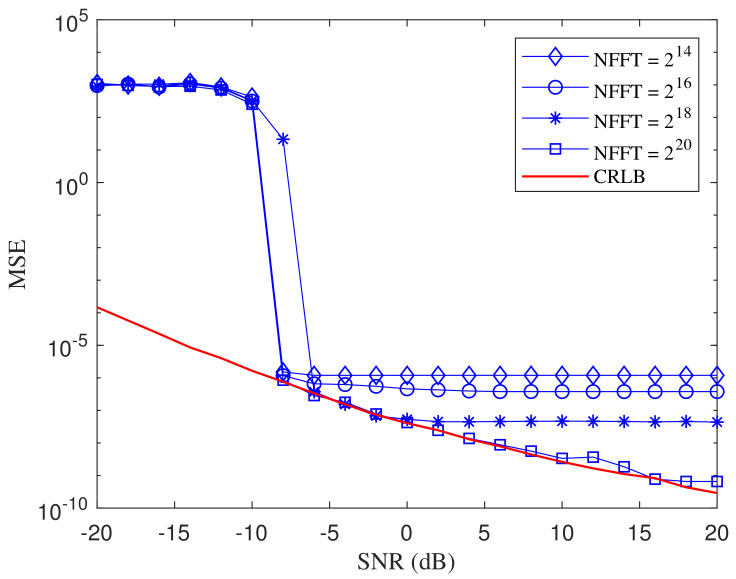
The MSE and the CRLB of frequency estimation with the proposed MLE for different NFFT.

Then the CRLB is expressed as $CRLB\left({\hat{f}}_{0}\right)=I^{-1}\left({\hat{f}}_{0}\right)$. Note that $s_{m}$ and $D_{m}$ are random variables in the expression of $CRLB({\hat{f}}_{0})$, where $s_{m}D_{m}$ is uniformly distributed in [−1,0], and $D_{m}^{2}$ is uniformly distributed in [0,1]. Hence, the average of CRLB is determined despite of the existence of the random variables. Figure 4 shows the comparison of the minimum square error (MSE) of the proposed MLE with a different number of FFT points (NFFT). The CRLB is depicted as the reference. The basis frequency $f_{0}$ is set as 1.12 Hz and the sampling frequency $f_{s}$ is set as 100 Hz. The time length of the test signal is $M/f_{0}$, where *M* is set as 100. As NFFT increases, the estimation error gradually approaches the theoretical lower bound.

## 4. Symbol Detection with a Difference-Integral Detector

At the friendly receiver of ASCS, the symbols are recovered with a coherent matched-filter-based detector which requires precise knowledge of the frequency parameter *f* and the damping coefficient β. However, the parameter β, which is related to the linear part of the ASCS system, is difficult to estimate since the received signal is severely contaminated by noise. In this section, we propose a DI detector to incoherently retrieve the binary symbols that the chaotic signal is conveying, which requires only the frequency parameter estimation.

**Figure 5:**
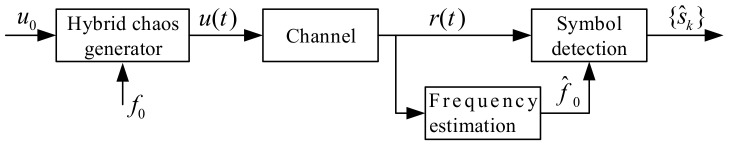
The block diagram of the frequency estimation and symbol detection.

The whole process of the frequency estimation and symbol detection is described in Figure 5. The chaotic signal is generated by the ASCS oscillator with the initial value $u_{0}$ and the basis frequency $f_{0}$. After transmitting in the channel, the chaotic signal is contaminated by the noise. Then we conduct a blind frequency estimation from the received signal and obtain the estimated basis frequency ${\hat{f}}_{0}$. With the estimated frequency ${\hat{f}}_{0}$, the binary symbols that the chaotic signal conveying can be retrieved by a DI detector.

As mentioned above, the main characteristic of ASCS is that this class of chaotic signal can be described by a weighted sum of basis functions. Note that continuous waveform u(t) contains the relations among binary symbols. Hence, the symbols conveyed in the ASCS waveform can be retrieved directly from the received signal by utilizing this feature.

**Figure 6:**
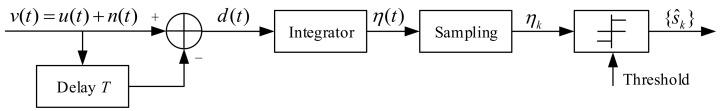
The block diagram of the DI -based detector.

The block diagram of the DI detector scheme is shown in Figure 6. Assume a segment of continuous chaotic signal u(t) carries a sequence of binary symbols $\left\{s_{k}\right\}$. The intermediate state d(t) is obtained by differencing the received signal v(t) and the delayed one v(t−T). Then d(t) is sent to the integrator. The integral output η(t−T) is computed numerically by $\int_{-\infty }^{t}d\left(t+\tau \right)d\tau$. Note that the practical signal generated from the chaotic oscillator is not infinite in length. Suppose the received signal transmit *N* symbols and thus the time duration is NT. Then the whole integral region is from 0 to NT. By extracting the integral output at sampling time $t_{k}=kT\left(k=0,1,\ldots ,N-1\right)$, we compare the samples $\eta_{k}$ with a flexible threshold $\theta_{k}$, calculated with the previously recovered symbols to detect the polarity, which is given by $\theta_{k}=-\lambda AB\sum_{m=0}^{k-1}s_{m}e^{-m\beta T}$. If the $\eta_{k}$ is larger than $\theta_{k}$, then the estimated symbol is 1. Otherwise, the estimated symbol is −1. Hence, the estimated symbol sequence is expressed as ${\hat{s}}_{k}=\mathrm{sgn}\left\{\eta_{k}-\theta_{k}\right\}$. In the following, we provide detailed derivations of the proposed detector.

The received signal in the AWGN channel is modeled as

(23) r(t)=u(t)+n(t)

where the white Gaussian noise term n(t) has zero mean and power density $N_{0}/2$. The difference between the received signal and its time-shift version can be expressed in a convolution form as

(24)d(t)=v(T)−v(t−T)(25)=∑m=0∞sm(P(t−mT)−P(t−T−mT))+n(t)−n(t−T).

Define an integral from 0 to *t* as

(26)η(t)=∫0td(τ+T)dτ(27)=∫0t∑m=0∞sm(P(t+T−mT)−P(t−mT))dτ+∫0t(n(τ+T)−n(τ))dτ(28)=ϵ(t)+χ(t)

where χ(t) is noise term; ϵ(t) is computed as

(29) $$\begin{array}{cc} \epsilon (t)= & -\lambda AB\sum_{m=0}^{k-2}s_{m}e^{-m\beta T}+s_{k-1}\left(-AB+kT-t+\frac{1}{\omega }e^{\beta (t-kT)}\sin\left(\omega t+\varphi \right)-\lambda ABe^{-(k-1)\beta T}\right)\\ & +s_{k}\left(AB+t-\left(k-1\right)T-\lambda ABe^{-k\beta T}+\frac{1}{\omega }\left(e^{-\beta T}-2\right)e^{\beta (t-kT)}\sin\left(\omega t+\varphi \right)\right)\\ & +\lambda \sum_{m=k+1}^{\infty }s_{m}\left(\frac{1}{\omega }e^{\beta (t-mT)}\sin\left(\omega t+\varphi \right)-e^{-m\beta T}AB\right) \end{array}$$

where $\lambda =e^{\beta T}+e^{-\beta T}-2$; $A=\frac{\beta^{2}}{\omega^{2}+\beta^{2}}$; $B=\frac{2}{\beta }$; $\varphi =\arctan\frac{2\beta \omega }{\omega^{2}-\beta^{2}}.$ To guarantee the accuracy of the integral, the integral step should be small and thus the sampling rate of the received signal v(t) is supposed to be high. The sample of ε(t) at t=kT is derived as

(30) $$\begin{array}{cc} \varepsilon_{k}= & -\lambda AB\sum_{m=0}^{k-1}s_{m}e^{-m\beta T}\\ & +s_{k}\left(-\lambda ABe^{-k\beta T}+\left(e^{-\beta T}-1\right)AB+T\right)\\ & +\lambda AB\left(e^{k\beta T}-1\right)\sum_{m=k+1}^{\infty }s_{m}e^{-m\beta T}. \end{array}$$

where the upper term is the cumulative interference caused by the previous symbols; the bottom term is the interference caused by the future symbols. Hence, we can rewrite the samples as

(31) $$\eta_{k}=s_{k}\left(-\lambda ABe^{-k\beta T}+\left(e^{-\beta T}-1\right)AB+T\right)+\Sigma +\xi$$

where ξ is the sampled noise term χ(t) in Equation (28 ) and it follows a Gaussian distribution with zero mean and $\sigma_{\xi }^{2}$ variance; Σ is the inter-symbol interference defined as

(32) $$\Sigma =I_{p}+I_{f}$$

where $I_{p}=-\lambda AB\sum_{m=0}^{k-1}s_{m}e^{-m\beta T}$ represents the intersymbol interference of the past symbols; $I_{f}=\lambda AB\left(e^{k\beta T}-1\right)\sum_{m=k+1}^{\infty }s_{m}e^{-m\beta T}$ represents the intersymbol interference of the future symbols. Since $I_{p}$ can be calculated but $I_{f}$ is unavailable, we use $I_{p}$ as the detection threshold and the detection process is described as

(33) $${\hat{s}}_{k}=\mathrm{sgn}\left\{\eta_{k}-\theta_{k}\right\}$$

with $\theta_{k}=I_{p}$ as the threshold.

**Figure 7:**
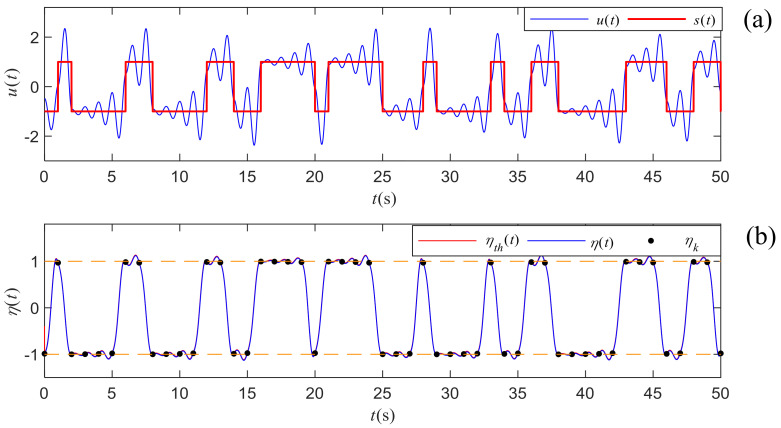
(**a**) The continuous waveform u(t) of ASCS and the corresponding binary symbol sequence s(t). (**b**) Numerical integral η(t) and the theoretical integral $\eta_{th}\left(t\right)$ computed by Equation (29). The dots are $\eta_{k}$ obtained by sampling the continuous integral result η(t) with a fixed sampling period of *T*.

To illustrate the detection process above, we generate a segment of chaotic signal with *m* truncated at 50 and the period *T* is set as 1 s, where the chaotic signal is not contaminated by noise. The continuous waveform and symbol sequence are depicted in Figure 7a. Both the numerical η(t) and theoretical $\eta_{th}\left(t\right)$ integrals are calculated and compared in Figure 7b, where the sampling rate is 0.01 s. From the comparison of the waveforms, we find that the theoretical integral computed by Equation (29) is quite close to the numerical integral result, proving that the previous calculation and approximation are reasonable. The samples $\eta_{k}$ of the numerical integral result η(t) stay around ±1 which are depicted with two horizontal dash lines. Hence, the symbol sequence can be easily retrieved by Equation (33). Compared with the original symbol sequence provided in Figure 7a, the extracted one is exactly the same.

## 5. Performance Evaluation and Simulation

In this section, we first evaluated the symbol error performance theoretically and derived an exact closed-form expression of error probability in the symbol detection. Then the robustness of the DI detector was verified by numerical simulations with various errors in parameter estimation.

Under the noise background, errors in symbol detection occur with rising probability as the variance of noise increases. Hence, it is necessary to evaluate the error performance for symbol detection with different SNR levels. In this paper, we define the BER as the ratio of the number of errors to the total number of detected symbols. As implied in Equation (33), the detection is a classical hypothesis test. Then the probability of detecting an incorrect bit is

(34) $$P_{e}=P\left(\eta_{k}>\theta_{k}|s_{k}=-1\right)P\left(s_{k}=-1\right)+P\left(\eta_{k}<\theta_{k}|s_{k}=1\right)P\left(s_{k}=1\right).$$

Note that the source symbols $\left\{s_{k}\right\}$ are sent with equal probability, that is, $P\left(s_{k}=-1\right)=P\left(s_{k}=1\right)=1/2$. Assume that “+1” and “−1” in the future symbols distribute with the equal probability of 1/2, then $I_{f}$ follows a uniform distribution in the range given by $\left[-\frac{\lambda AB(1-e^{-k\beta T})}{e^{\beta T}-1},\frac{\lambda AB(1-e^{-k\beta T})}{e^{\beta T}-1}\right]$. Note that the term $e^{-k\beta T}$ decreases rapidly as *k* increases, then we have the approximate upper bound as $-\frac{\lambda AB}{e^{\beta T}-1}$ and the upper bound as $\frac{\lambda AB}{e^{\beta T}-1}$. Thus, the error probability for $s_{k}=1$ can be expressed as

(35) $$P_{e}=\int_{-\frac{\lambda AB}{e^{\beta T}-1}}^{\frac{\lambda AB}{e^{\beta T}-1}}\frac{1}{2}\mathrm{erfc}\left(\frac{\Phi +I_{f}}{\sqrt{2}\sigma_{\xi }}\right)f\left(I_{f}\right)dI_{f}.$$

where erfc(·) denotes the complementary error function; $\Phi =-\lambda ABe^{-k\beta T}+\left(e^{-\beta T}-1\right)AB+T$. Consider that $e^{-k\beta T}$ decreases rapidly as *k* increases, we have the approximate $\Phi =(e^{-\beta T}-1)AB+T$. The integral is computed as

(36) $$P_{e}=\sqrt{2\sigma_{\xi }^{2}}\frac{e^{\beta T}-1}{4\lambda AB}\left(z_{1}\mathrm{erfc}\left(z_{1}\right)-z_{2}\mathrm{erfc}\left(z_{2}\right)-\frac{1}{\sqrt{\pi }}e^{-z_{1}^{2}}+\frac{1}{\sqrt{\pi }}e^{-z_{2}^{2}}\right).$$

where $z_{1}=\frac{1}{\sqrt{2}\sigma_{\xi }}\left(\Phi +\frac{\lambda AB}{e^{\beta T}-1}\right)$; $z_{2}=\frac{1}{\sqrt{2}\sigma_{\xi }}\left(\Phi -\frac{\lambda AB}{e^{\beta T}-1}\right)$.

**Figure 8:**
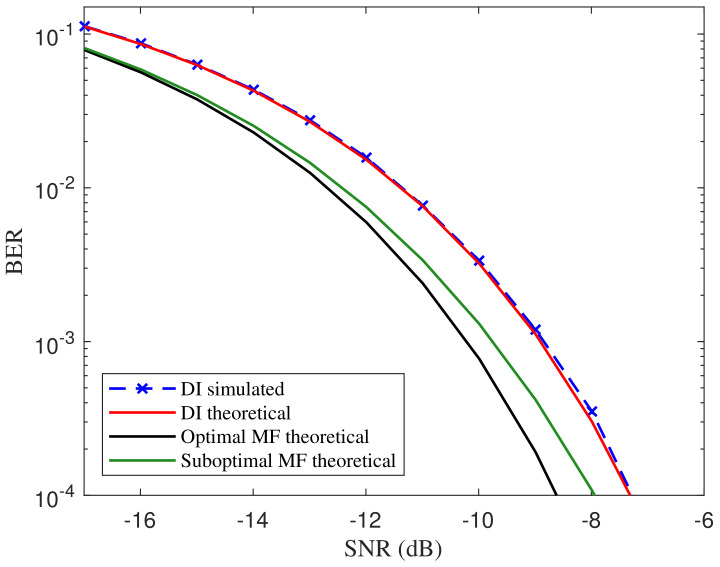
BER comparison of the proposed DI detector scheme to the optimal matched filter, suboptimal matched filter for f=1 Hz, β=ln2 and sampling frequency as $T_{s}=0.01$ s.

We implement our symbol detecting method in different frequency settings and illustrate the BER curves in Figure 8. The theoretical BER curves of the symbol detecting method with optimal and suboptimal matched filter introduced in References [4,16] are also depicted as a reference. The SNR is defined as the ratio of the power of the ASCS signal to the noise power, where the sample rate is 100 times faster than the symbol rate. In fact, the relative SNR calculated with in-band noise should be the actual SNR added to $10\log_{10}50$ dB. As can be noticed, the simulated curve for the proposed DI detector matches the theoretical one well. Compared with the theoretical error performance of the suboptimal matched-filter-based detector, our method performs a degradation around 1 dB. Nevertheless, it is acceptable for an attacker or an eavesdropper to obtain enough information.

**Figure 9:**
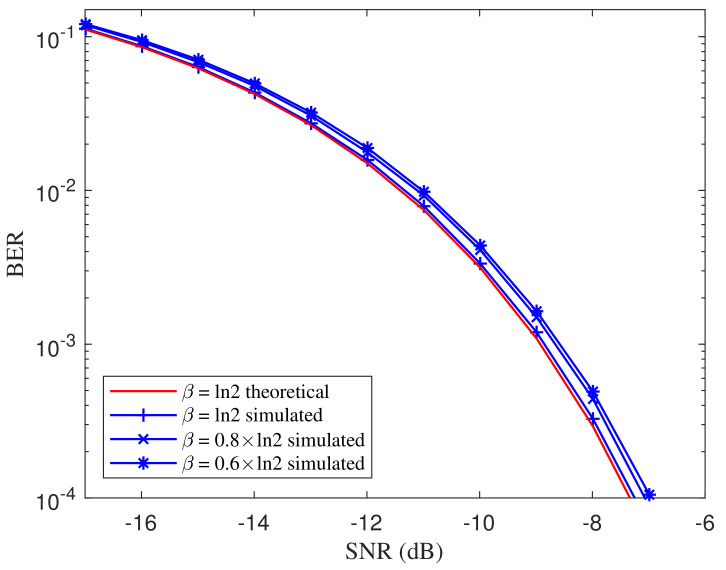
BER comparison of the proposed DI detector over different damping coefficients β=afln2. The basis frequency is set as 1 Hz. The coefficient *a* is in {0.6,0.8,1}.

**Figure 10:**
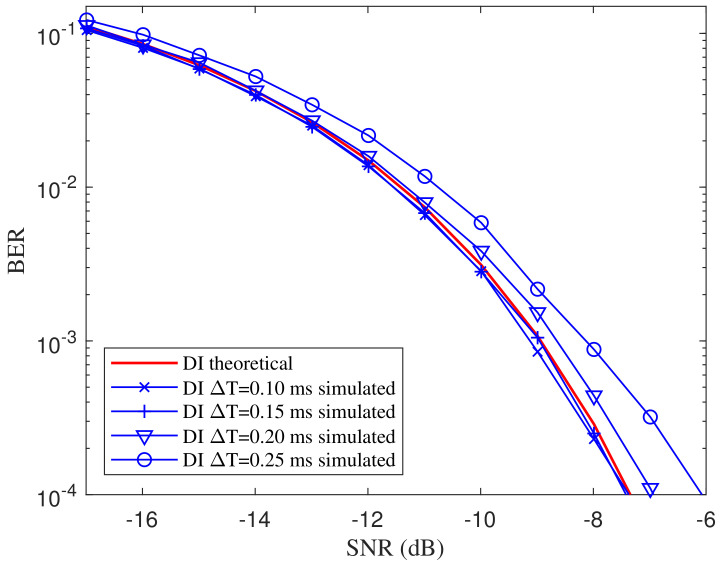
BER comparison of the proposed DI detector over different sampling lag ΔT.

As implied in the theoretical BER expression Equation (36), the BER is related to the damping coefficient β. To evaluate the BER performance under different damping coefficients, we set the damping coefficient as β=afln2 where the coefficient a∈{0.6,0.8,1}. The simulated BER curves of the proposed DI-HCC over different damping coefficients are shown in Figure 9. The theoretical BER for a=1 is depicted as a reference. From the comparisons of BER curves over different settings, the BER performance of the DI detector slightly degrades as the damping coefficient β decreases. However, the deterioration is not significant since β has a limited impact on the constant values in Equation (36). Hence, the proposed DI detector is robust to the damping coefficient of the ASCS. Since the samples are extracted at the integer multiplies of *T*, the imprecise sampling time is supposed to have effects on the BER performance. Assume that the integral η(t) are sampled at t=kT+ΔT, where ΔT is the sampling lag. Figure 10 shows the BER curves over different sampling lags with ΔT∈{0.10,0.15,0.20,0.25} ms where the basis frequency of the ASCS is set as 1 kHz. From the comparison of the curves, the performance deteriorates gradually as the lag increases. However, the deterioration is slight when the lag is less than 0.15 ms, meaning the proposed detector keeps valid within a limited range of sampling lag.

**Figure 11:**
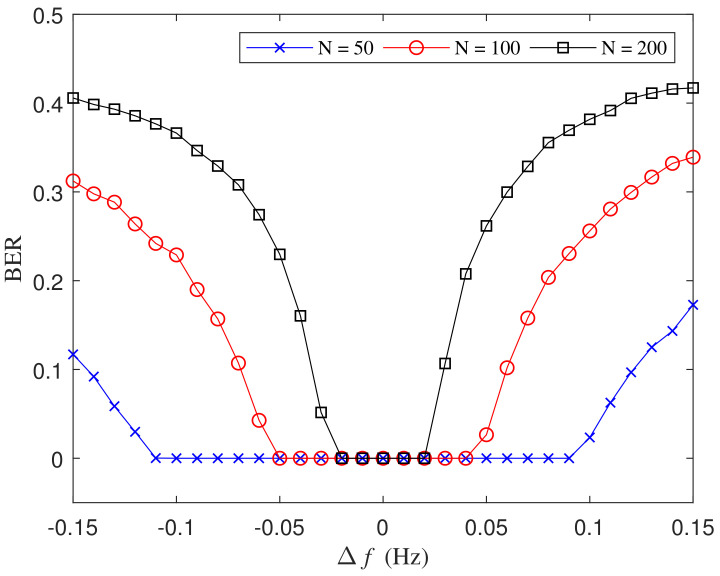
BER comparison for different number of symbols conveying in chaotic signal.

The key parameter in symbol detection is the symbol period *T*, which is defined as $T=1/f_{0}$ and the basis frequency $f_{0}$ is estimated by the FFT-based MLE. Unavoidably, the frequency estimation error has a negative impact on the symbol detection. Figure 11 illustrates BERs for various estimation error Δf with different numbers of symbols in the received signal with a fixed SNR as −5 dB, where the basis frequency is set as $f_{0}=10$ Hz and the estimated frequency is $f_{0}+\Delta f$. As the absolute value of the frequency estimation error increases, the symbol detection error probability is larger when *N* is 200 while almost no error is seen for N=50 and N=100 in the tested estimation error range. It can be explained that the DI detection is a process with error accumulation over time. As time passes, the sampling time will gradually deviate from the original exact sampling positions, which brings deterioration to the symbol detection. Assume the difference between the estimated symbol period $T^{\prime }=1/\left(f_{0}+\Delta f\right)$ and the exact period *T* is $\Delta T=T^{\prime }-T$. Suppose the error of sampling time increases to the half of *T* after *N* symbols, then we have NΔT=T/2 and the estimation error of the frequency is correspondingly Δf=f/(2N−1). Hence, the maximum of the absolute value of the frequency estimation error should be less than Δf. In the simulation in Figure 11, as derived theoretically, the absolute value of Δf should be controlled within 0.025, 0.05, and 0.1 for *N* is set as 200, 100, 50 respectively, to avoid the accumulated error. The obtained BERs show that the theoretical bounds of the frequency estimation error for different *N* match the simulated results well. In the practical implementation of the proposed method, the frequency estimation error can be evaluated by CRLB with the known SNR level. With the theoretical frequency estimation error, we can compute the upper bound of *N*. Then the received signal is divided into segments with the limited time length of NT to avoid error caused by the accumulation effect.

## 6. Conclusions

In this paper, an efficient method of blind frequency estimation and symbol recovery for ASCS is proposed. To obtain the frequency parameter of the chaotic signal, we first proposed a blind frequency estimator based on the square power of the received signal. Then we designed a DI detector by integrating the difference between the received signal and its time-delayed version. The binary symbols conveyed in the chaotic signal can be retrieved directly from the sampled integral results. The theoretical analysis of the proposed scheme is provided with detailed derivations and performance evaluation. For the frequency estimation, the CRLB is derived to assess the lower bound on the error variance. For the symbol detection, the close-form BER expression is derived to evaluate the error in symbol detection. Moreover, the robustness of our proposed method in various parameter settings is verified through numerical experiments. Both the theoretical derivation and simulations prove that the proposed method performs well against strong noise. 

## Appendix A. Derivation of the Amplitude Spectrum of the Chaotic Signal

The integral of the Equation (12) is computed as

(A1) $$\begin{array}{c} b\left(\omega \right)=A\sum_{m=-\infty }^{\infty }s_{m}D_{m}\\ \times (\frac{\beta \sin\theta_{m}-\left(\omega_{0}-\omega \right)\cos\theta_{m}}{\beta^{2}+{\left(\omega_{0}-\omega \right)}^{2}}+\frac{-\beta \sin\lambda_{m}+\left(\omega_{0}+\omega \right)\cos\lambda_{m}}{\beta^{2}+{\left(\omega_{0}+\omega \right)}^{2}}\\ +e^{\beta T}\left(\frac{-\beta \sin\delta_{m}+\left(\omega_{0}-\omega \right)\cos\delta_{m}}{\beta^{2}+{\left(\omega_{0}-\omega \right)}^{2}}+\frac{\beta \sin\eta_{m}-\left(\omega_{0}+\omega \right)\cos\eta_{m}}{\beta^{2}+{\left(\omega_{0}+\omega \right)}^{2}}\right)). \end{array}$$

When $\omega \neq n\omega_{0}$, $\sum_{m=-\infty }^{\infty }\theta_{m}\to 0$ since $\theta_{m}$ is ergodic in −∞ to *∞*. When $\omega =n\omega_{0}$, the variables $\theta_{m}$, $\lambda_{m}$, $\delta_{m}$, and $\eta_{m}$ keep constant since mTω=2mnπ.

The Fourier transform for $w_{2}\left(t\right)$ is computed as

(A2) $$\begin{array}{cc} W_{2}\left(\omega \right) & =\int_{-\infty }^{\infty }w_{2}\left(t\right)e^{-j\omega t}dt\\ & =\frac{A^{2}}{2}\sum_{m=-\infty }^{\infty }D_{m}^{2}\int_{mT}^{(m+1)T}e^{2\beta (t-mT)}\left(\cos\left(2\omega_{0}t+2\varphi \right)+1\right)e^{-j\omega t}dt. \end{array}$$

We have the coefficients of Fourier transform as

(A3) $$p\left(\omega \right)=\frac{A^{2}}{2}\sum_{m=-\infty }^{\infty }D_{m}^{2}\int_{mT}^{(m+1)T}e^{2\beta (t-mT)}\left(\cos\left(2\omega_{0}t+2\varphi \right)+1\right)\cos\omega tdt$$

and

(A4) $$q\left(\omega \right)=\frac{A^{2}}{2}\sum_{m=-\infty }^{\infty }D_{m}^{2}\int_{mT}^{(m+1)T}e^{2\beta (t-mT)}\left(\cos\left(2\omega_{0}t+2\varphi \right)+1\right)\sin\omega tdt.$$

By caculating the integrals above, we have

(A5) $$\begin{array}{c} p\left(\omega \right)=\frac{A^{2}}{4}\sum_{m=-\infty }^{\infty }D_{m}^{2}\\ \times (\frac{-4\beta \cos\nu_{m}-2\omega \sin\nu_{m}}{4\beta^{2}+\omega^{2}}+\frac{-2\beta \cos\gamma_{m}+\left(-2\omega_{0}+\omega \right)\sin\gamma_{m}}{4\beta^{2}+{\left(-2\omega_{0}+\omega \right)}^{2}}\\ -\frac{2\beta \cos\varsigma_{m}+\left(2\omega_{0}+\omega \right)\sin\varsigma_{m}}{4\beta^{2}+{\left(2\omega_{0}+\omega \right)}^{2}}+e^{2\beta T}(\frac{4\beta \cos\upsilon_{m}+2\omega \sin\upsilon_{m}}{4\beta^{2}+\omega^{2}}\\ +\frac{2\beta \cos\kappa_{m}+\left(2\omega_{0}-\omega \right)\sin\kappa_{m}}{4\beta^{2}+{\left(2\omega_{0}-\omega \right)}^{2}}+\frac{2\beta \cos\iota_{m}+\left(2\omega_{0}+\omega \right)\sin\iota_{m}}{4\beta^{2}+{\left(2\omega_{0}+\omega \right)}^{2}})) \end{array}$$

and

(A6) $$\begin{array}{c} q\left(\omega \right)=\frac{A^{2}}{4}\sum_{m=-\infty }^{\infty }D_{m}^{2}\\ \times (\frac{-4\beta \sin\nu_{m}+2\omega \cos\nu_{m}}{4\beta^{2}+\omega^{2}}+\frac{2\beta \sin\gamma_{m}+\left(-2\omega_{0}+\omega \right)\cos\gamma_{m}}{4\beta^{2}+{\left(-2\omega_{0}+\omega \right)}^{2}}\\ +\frac{-2\beta \sin\varsigma_{m}+\left(2\omega_{0}+\omega \right)\cos\varsigma_{m}}{4\beta^{2}+{\left(2\omega_{0}+\omega \right)}^{2}}+e^{2\beta T}(\frac{4\beta \sin\upsilon_{m}-2\omega \cos\upsilon_{m}}{4\beta^{2}+\omega^{2}}\\ -\frac{2\beta \sin\kappa_{m}-\left(2\omega_{0}-\omega \right)\cos\kappa_{m}}{4\beta^{2}+{\left(2\omega_{0}-\omega \right)}^{2}}+\frac{2\beta \sin\iota_{m}-\left(2\omega_{0}+\omega \right)\cos\iota_{m}}{4\beta^{2}+{\left(2\omega_{0}+\omega \right)}^{2}})) \end{array}$$

where $\nu_{m}=mT\omega$; $\gamma_{m}=2\varphi -mT\omega$; $\varsigma_{m}=2\varphi +mT\omega$; $\upsilon_{m}=\left(1+m\right)T\omega$; $\kappa_{m}=2\varphi -\left(1+m\right)T\omega$; $\iota_{m}=2\varphi +\left(1+m\right)T\omega$. With the ergodicity of these variables for $\omega \neq n\omega_{0}$, the cumulative sums of p(ω) and q(ω) over m∈(−∞,∞) are close to zero. For $\omega =n\omega_{0}$, the variables are constant and we obtain the amplitude spectrum of $W_{2}\left(\omega \right)$ as

(A7) $$|W_{2}\left(n\omega_{0}\right)|=\frac{2A^{2}\left(e^{2\beta T}-\frac{1}{2}\right)}{\beta^{2}+\omega_{0}^{2}}\sum_{m=-\infty }^{\infty }D_{m}^{2}\sqrt{\frac{100\beta^{4}\omega_{0}^{4}+8\left(5+2n^{2}\right)\beta^{2}\omega_{0}^{6}+{\left(n^{2}-2\right)}^{2}\omega_{0}^{8}}{\left(4\beta^{2}+{\left(-2+n\right)}^{2}\omega_{0}^{2}\right)\left(4\beta^{2}+n^{2}\omega_{0}^{2}\right)\left(4\beta^{2}+{\left(2+n\right)}^{2}\omega_{0}^{2}\right)}}$$

For $\omega \neq n\omega_{0}$, we have

(A8) $$|W_{2}\left(\omega \right)|=\sqrt{p^{2}\left(\omega \right)+q^{2}\left(\omega \right)}.$$
